# Influential Nodes Identification in the Air Pollution Spatial Correlation Weighted Networks and Collaborative Governance: Taking China’s Three Urban Agglomerations as Examples

**DOI:** 10.3390/ijerph19084461

**Published:** 2022-04-07

**Authors:** Feipeng Guo, Zifan Wang, Shaobo Ji, Qibei Lu

**Affiliations:** 1 School of Management and E-Business, Zhejiang Gongshang University, Hangzhou 310018, China; 21020200029@pop.zjgsu.edu.cn; 2 Modern Business Research Center, Zhejiang Gongshang University, Hangzhou 310018, China; 3 Sprott School of Business, Carleton University, Ottawa, ON K1S 5B6, Canada; shaobo.ji@carleton.ca; 4 School of International Business, Zhejiang International Studies University, Hangzhou 310023, China; luqibei@zisu.edu.cn

**Keywords:** influential nodes identification, air pollution, complex network, China’s three major urban agglomerations, carbon peak and carbon neutrality, collaborative governance

## Abstract

Nowadays, driven by green and low-carbon development, accelerating the innovation of joint prevention and control system of air pollution and collaborating to reduce greenhouse gases has become the focus of China’s air pollution prevention and control during the “Fourteenth Five-Year Plan” period (2021–2025). In this paper, the air quality index (AQI) data of 48 cities in three major urban agglomerations of Beijing-Tianjin-Hebei, Pearl River Delta and Yangtze River Delta, were selected as samples. Firstly, the air pollution spatial correlation weighted networks of three urban agglomerations are constructed and the overall characteristics of the networks are analyzed. Secondly, an influential nodes identification method, local-and-global-influence for weighted network (W_LGI), is proposed to identify the influential cities in relatively central positions in the networks. Then, the study area is further focused to include influential cities. This paper builds the air pollution spatial correlation weighted network within an influential city to excavate influential nodes in the city network. It is found that these influential nodes are most closely associated with the other nodes in terms of spatial pollution, and have a certain ability to transmit pollutants to the surrounding nodes. Finally, this paper puts forward policy suggestions for the prevention and control of air pollution from the perspective of the spatial linkage of air pollution. These will improve the efficiency and effectiveness of air pollution prevention and control, jointly achieve green development and help achieve the “carbon peak and carbon neutrality” goals.

## 1. Introduction

During the “Fourteenth Five-Year Plan” period (2021–2025), in order to continuously improve China’s air quality and reduce greenhouse gases, ecological environmental protection will move from pollutant management to collaborative governance of pollution reduction and carbon reduction, which is an important turning point for China’s air pollution prevention and control and a milestone for the deep integration of pollution prevention and control with “carbon peak and carbon neutrality” [1]. At present, air quality monitoring stations are built in each city, which continuously monitor the following pollutants in the air for 24 h: PM_2.5_, PM_10_, SO_2_, CO, NO_2_, O_3_, etc. These pollutants have different degrees of impact on the air quality of the cities. Analysis of air pollution characteristics has been a very hot topic at home and abroad. Scholars have studied the causes [2,3], influencing factors [4], monitoring and analysis of pollutants [5,6], spatial and temporal characteristics [7,8], and transmission paths [9,10,11] of air pollution. The results of the above studies all show that air pollution is characterized by an obvious spatial correlation. However, due to the limitations of sample data and research methods, these existing studies lack further exploration of the spatial correlation of air pollution.

Urban agglomerations are an important engine for raising the level of urbanization and maintaining sustainable high-quality economic development in China. With the rapid development of urban agglomerations, China’s economic aggregate has grown rapidly. However, it has also brought a series of ecological and environmental problems [12]. Currently, the level of air pollution in the Beijing-Tianjin-Hebei, the Pearl River Delta, the Yangtze River Delta and other urban agglomerations with relatively high levels of economic development is significantly higher than the national level [13]. Due to the cross-influence of air pollution in adjacent cities, air pollution in a certain area is not only related to local social production and people’s lives but is also affected by the ecological environment of surrounding areas [14] and the dynamic spatial correlation between multiple areas constitutes a complex network. At present, research applying complex network theory to air pollution has been increasing. Some scholars at home and abroad have studied the spatial correlation characteristics of air pollution from the perspectives of network topology characteristics analysis [15] and pollutant dynamic behavior analysis [16]. However, due to geographical location, wind direction, and other factors, the degree of interconnection and interaction of air pollution between regions is not consistent and the nodes in the network belong to the core and edge positions [17,18]. Therefore, applying the influential nodes identification method to the study of the spatial correlation of air pollution can help identify the real influential nodes and dig out small areas with serious pollution and strong transmission ability so that more targeted measures can be taken to control and protect.

This paper takes 48 cities in the three major urban agglomerations of Beijing-Tianjin-Hebei, Pearl River Delta and Yangtze River Delta as the study areas. We use the AQI daily average data of each city and air quality monitoring station during a cascade year from 1 November 2020 to 31 October 2021 to construct the air pollution spatial correlation weighted networks of the three major urban agglomerations and analyze the network density, network efficiency, and network rank degree of each network. Since the weighted network can describe the closeness of the air pollution correlation between the node cities and express the structure of the complex network more realistically and in detail, this paper proposes a method for the identification of influential nodes, local-and-global-influence for weighted network (W_LGI), to evaluate the influence of each node in the air pollution spatial correlation weighted networks and to identify the influential node cities that are relatively core in the urban agglomeration networks. The study area is then refined to the influential city. We build the air pollution spatial correlation weighted network within influential cities and uncover the influential nodes in the city network—the air quality monitoring stations. Finally, based on the above analysis results, we propose countermeasures for air pollution prevention and control, providing new ideas for prevention and control from the perspective of spatial correlation of air pollution.

The structure of this paper is as follows. The second part introduces the related works of the study. The third part introduces the research methods, including the construction method of air pollution spatial correlation weighted network, the analysis method of network topology characteristics, and the influential nodes identification method based on weighted network. The fourth part is the experimental analysis, constructing the air pollution spatial correlation weighted networks of three major urban agglomerations and one influential city, and evaluating the influences of the nodes in the air pollution spatial correlation weighted networks. Finally, the conclusions and suggestions are presented in the fifth part.

## 2. Related Works

Since the publication of the seminal work of the WS small-world model [19] and the BA scale-free model [20], complex network theory has been widely used in many fields, such as sociology, natural science, management science, and ecology, etc. It is a notable trend in recent years to introduce complex system and complex network theory into the study of social governance. Many scholars use the tool of complex networks to describe complex systems. A typical network consists of nodes and the edges connecting them, where the nodes represent the different individuals in a complex system and the edges represent the relationships between individuals. Scholars currently use complex networks to describe complex systems in different fields, such as social networks [21], transportation networks [22], biological networks [23], stock networks [24], etc. There are relatively few studies that apply complex networks to environmental science and in particular to air pollution. Relevant studies have been conducted mainly at the national, urban agglomeration, and single city levels. Studies at the national and urban agglomeration levels take cities as nodes of the network, while at the single city level, air quality monitoring stations are used as nodes of the network. At the national level, Jin et al. [25] analyzed the spatial correlation characteristics of PM_2.5_ emissions in China and applied a network partitioning algorithm to find that PM_2.5_ emissions between provinces have significant mutual effects; Broomandi et al. [26] analyzed PM_2.5_ concentrations in 14 cities in the UK and roughly divided the UK into two connected regions, the south and the north, by constructing a Granger causal network. At the urban agglomeration level, Li et al. [27] proposed a joint regional air pollution prevention and control method for the Beijing-Tianjin-Hebei region and they believed that it is most cost-effective to carry out the same cluster control for the entire city; Ma et al. [28] used node importance mining to identify the closest sub-networks with the most frequent pollution relationships in the Beijing-Tianjin-Hebei region by constructing a weighted network. At the single city level, Zhang et al. [29] conducted a network topology analysis of the dynamic patterns of NO_2_ and O_3_ over time series in Lanzhou City to explore the correlation between the two pollutants. It can be seen that the advantage of applying complex network theory to the study of air pollution is that it can effectively describe the relationships and strength of the interactions of regional air pollution networks.

At present, scholars’ researches on air pollution using complex network theory mainly focus on the network topology, network structure, and statistical analysis of edges, and there are few studies on the identification of influential nodes. Due to geographical location, wind direction, and other factors, the degree of interconnection and interaction of air pollution between regions is not consistent. Current studies on the diffusion and transmission of air pollutants in the region mostly adopt the NAQPMS model [30], WRF-CHEM model [31], CAMx model [32], CMAQ model [33], etc. These models have very strict requirements on the quality of basic data, so it is difficult to accurately estimate air quality in local areas. The identification of influential nodes in complex networks can identify nodes that play an important role in the network structure and information transmission process [34], so applying the influential nodes identification method to the study of spatial correlation of air pollution can help find out the real influential nodes so that more targeted measures can be taken for governance and protection. A large number of metrics and algorithms have been proposed to identify influential nodes in the network: algorithms based on information about neighbor nodes (such as Degree Centrality [35], Semi-local Centrality [36], etc.) evaluate the importance of nodes through its local information; algorithms based on network information dissemination paths (such as Closeness Centrality [37], Betweenness Centrality [38], etc.) consider the position of nodes on the information dissemination path; algorithms based on the rank of nodes position (such as k-shell algorithm [39], etc.) consider the position of nodes in the global structure of the network; algorithms based on eigenvector (such as PageRank [40], LeaderRank [41], Eigenvector Centrality [42], etc.) not only consider the number of neighbor nodes but also the importance of them. Each of these methods has its own advantages and disadvantages, and it is important to choose an appropriate algorithm for a specific application scenario.

In summary, the application of complex network theory to the field of air pollution has attracted extensive research by scholars at home and abroad. However, these studies mainly focus on network topology, network structure, and statistical analysis of edges, and there are still fewer studies on the identification of influential nodes. In addition, there are limitations to current methods for influential nodes identification: the local discovery methods lack the consideration of the overall information of the network, and the global discovery methods have a relatively large time complexity. Therefore, this paper proposes an influential nodes identification method, local-and-global-influence for weighted network (W_LGI), which utilizes not only local information of nodes but also global structural information, and applies it to the study of spatial correlation of air pollution, providing new ideas for the collaborative governance of air pollution and the realization of the “carbon peak and carbon neutrality” goals.

## 3. Method

**Figure 1:**
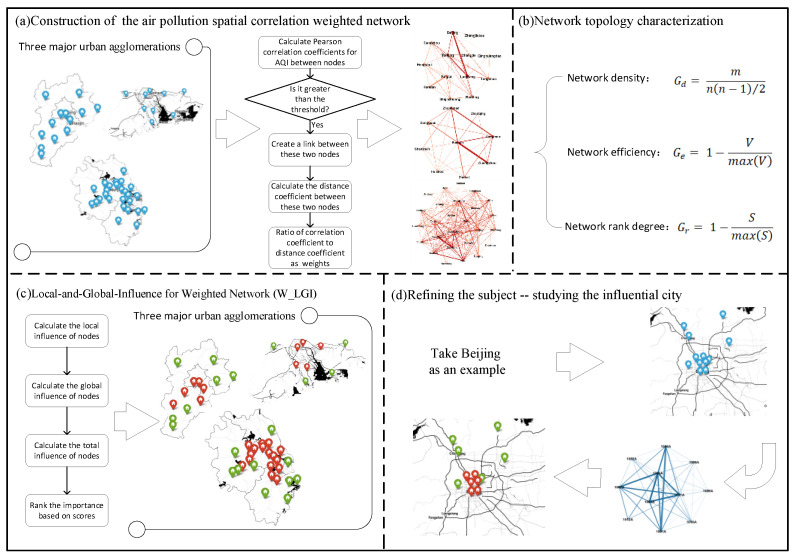
Flow chart of influential nodes identification method for air pollution spatial correlation weighted networks.

This paper takes the Beijing-Tianjin-Hebei, Pearl River Delta, and Yangtze River Delta urban agglomerations as the study areas, and uses the Pearson correlation coefficient of the daily average AQI data among cities as the criteria for whether the cities are connected to each other. If they are connected to each other, the ratio of the correlation coefficient to the distance coefficient is used as the weight of the edge to construct air pollution spatial correlation weighted networks of each urban agglomeration. By calculating the network density, network efficiency, and network rank degree of each network, the overall network characteristics of the spatial correlation of air pollution are reflected. On this basis, an improved influential nodes identification method combining local and global information is proposed to identify influential node cities in the networks. The study area is then refined, the air pollution spatial correlation weighted network within the influential city is constructed, and the influential nodes in the city network, the air quality monitoring stations, are identified. The overall method flow of this paper is shown in Figure 1.

### 3.1. Construction Method of the Air Pollution Spatial Correlation Weighted Network

A complex network is composed of a large number of nodes and edges, which can be represented in the form of a graph: G=(V, E, W), where V denotes the set of nodes, representing the individuals in the complex network; E denotes the set of edges, representing the connections between individuals; and W denotes the weight of the edge, which represents the strength of the connections between individuals. The adjacency matrix of a network represents the connection relationship between each node. For an undirected weighted network, the matrix element $w_{\mathrm{ij}}$ of the adjacency matrix can be expressed as:

(1) $$w_{\mathrm{ij}}=\{\begin{array}{cc} w_{\mathrm{ij}}, & \mathrm{If}\;\mathrm{node}\;i\;\mathrm{is}\;\mathrm{connected}\;\mathrm{to}\;j,\\ & w_{\mathrm{ij}}\;\mathrm{denotes}\;\mathrm{the}\;\mathrm{weight}\;\mathrm{of}\;\mathrm{the}\;\mathrm{edge}\\ 0, & \;\mathrm{If}\;\mathrm{nodes}\;i\;\mathrm{and}\;j\;\mathrm{are}\;\mathrm{not}\;\mathrm{connected} \end{array}$$

The air quality index (AQI) simplifies the detected air concentration into a single conceptual index value according to the proportion of various components in the air, including PM_2.5_, PM_10_, SO_2_, CO, NO_2_, O_3_ and other pollutants, which is suitable for analyzing air quality and its changing trend [43]. So, this paper uses AQI as a comprehensive index to measure air pollution. When constructing the air pollution spatial correlation weighted network in urban agglomerations, cities are used as nodes, and the Pearson correlation coefficient of the daily average value of AQI between cities and cities is used as the criterion for determining whether they are connected, where the Pearson correlation coefficient (as shown in Equation (2), where X and Y are two variables, respectively, and $X_{i}$ and $Y_{i}$ are the observed values of variable X and Y corresponding to i; $\bar{X}$ and $\bar{Y}$ are the average of X and Y samples) is often used to measure the correlation between two time-series variables with values between −1 and 1 [44]. In order to build a more stable network structure, this paper selects the mean value of all elements outside the diagonal of the correlation coefficient matrix as the threshold value. Such a threshold value selection method will make the change of the number of nodes of the maximum connected subgraph in the network more stable (that is, the topological nature of the network is stable) [45]. When the correlation coefficient is greater than the threshold, the two cities are connected. Otherwise no connection is established.

(2) $$r=\frac{\sum_{i=1}^{n}\left(X_{i}-\bar{X}\right)\left(Y_{i}-\bar{Y}\right)}{\sqrt{\sum_{i=1}^{n}{\left(X_{i}-\bar{X}\right)}^{2}}\sqrt{\sum_{i=1}^{n}{\left(Y_{i}-\bar{Y}\right)}^{2}}}$$

The pollutants in the air mainly originate from soil dust and fugitive dust, biomass combustion, automobile exhaust, etc. At the same time, they can also spread to other areas with air movement, transportation, industrial transfer and other activities. Since the transmission of pollutants in the air is also affected by factors such as distance between cities, terrain, and meteorological conditions, considering only the correlation coefficient cannot fully reflect the spatial relationship of the network. In order to better reflect the spatial relationship between different cities, this paper uses the ratio of the correlation coefficient of the daily average AQI values between cities and the ellipsoid distance between cities as the weight of the edges in the network structure [46], thus constructing a weighted network of spatial correlations of air pollution. In the actual calculation, this paper selects the linear proportional transformation method to eliminate the dimension of the distance between cities, divides the distance by the maximum distance to obtain a distance coefficient in the range of (0, 1], and again using the method to obtain the weights between (0, 1].

### 3.2. Analysis Method of Network Topology Characterization

In this paper, network density, network efficiency, and network rank degree are used to reflect the overall network characteristics of the spatial correlation of air pollution. The network density reflects the closeness of the network connection. As shown in Equation (3), the greater the network density, the closer the spatial connection of air pollution in urban agglomerations, and the correlation network structure has a stronger influence on air pollution. The network efficiency reflects the degree of redundant lines in the network. As shown in Equation (4), the lower the network efficiency, the more edges between node cities, the closer the connection, and the more stable the network structure. The network rank degree reflects the degree of asymmetric reachability of nodes. As shown in Equation (5), the higher the network rank degree, the stricter the hierarchical structure between node cities, and the more significant the asymmetric spatial spillover effect, with node cities belonging to core and edge positions [47].

(3) $$G_{d}=\frac{m}{n\left(n\;-\;1\right)/2}$$

(4) $$G_{e}=1\;-\frac{V}{\max\left(V\right)}$$

(5) $$G_{r}=1\;-\frac{S}{\max\left(S\right)}$$

In the equations, $G_{d}$, $G_{e}$, and $G_{r}$ represent network density, network efficiency and network rank degree, respectively, m represents the actual number of edges in the network, n represents the number of nodes, V. represents the number of redundant lines, and max(V) represents the maximum possible number of redundant lines, S. represents the number of symmetrically reachable node pairs in the network, and max(S) represents the maximum possible number of node pairs in the network.

### 3.3. Influential Nodes Identification Method—Local-and-Global-Influence for Weighted Network (W_LGI)

At present, a large number of methods have been proposed to identify influential nodes in complex networks. Traditional methods of finding influential nodes in complex networks can be divided into local discovery and global discovery. Local discovery only considers the node’s own attributes and its neighbor information, such as Degree Centrality, PageRank, etc.; and global discovery focuses on measuring the global information of nodes in the network, such as Betweenness Centrality, Closeness Centrality, etc. Although each of these methods has its own advantages, there are certain limitations. For example, the local discovery method lacks the overall information of the network, and the time complexity of the global discovery method is relatively large [48]. Therefore, this paper proposes an influential nodes identification method for undirected weighted networks called local-and-global-influence for weighted network (W_LGI), which not only considers the local structure of nodes in the network but also pays attention to the global structure to effectively mining influential nodes.

In an unweighted network, the degree of a node is the number of other nodes directly connected to the node; in a weighted network, the degree of a node is the sum of the weights of the edges directly connected to the node [49], as shown in Equation (6). The degree of a node can reflect the local structural information of the node, so the ratio of the weighted degree of the node to the total number of nodes in the network can be used to measure the local influence of a node, as shown in Equation (7).

(6) $$d_{w}\left(v_{i}\right)=\underset{j\in V}{\sum }w_{\mathrm{ij}}.\;$$

(7) $$W_{-}LI\left(V_{i}\right)=\frac{d_{w}\left(v_{i}\right)}{n}$$

In the equations, $d_{w}\left(v_{i}\right)$ represents the degree of the node $v_{i}$ considering the edge weights, $W\text{\_}\mathrm{LI}\left(v_{i}\right)$ represents the local influence of the node $v_{i}$, $w_{\mathrm{ij}}$ represents the edge weight between the nodes $v_{i}$ and $v_{j}$, and n represents the total number of nodes in the network.

The degree can reflect the edge status of the node itself but cannot reflect the edge status of the neighbor nodes. In fact, the node will also be affected by other nodes in the network. The shortest distance between a node and other nodes in the network can reflect the global influence of the node, which is inversely proportional to the influence of the node. Therefore, this paper uses the ratio of the weighted degree of nodes to the shortest distance between nodes to measure the global influence of a node, as shown in Equation (8).

(8) $$W\text{\_}\mathrm{GI}\left(v_{i}\right)=\underset{i\neq j}{\sum }\frac{\sqrt{d_{w}\left(v_{j}\right)}}{d_{\mathrm{ij}}+\theta }$$

In the equation, $W\text{\_}\mathrm{GI}\left(v_{i}\right)$ represents the global influence of node $v_{i}$, $d_{w}\left(v_{j}\right)$ represents the weighted degree of node $v_{j}$, $d_{\mathrm{ij}}$ represents the shortest distance between node $v_{i}$ and $v_{j}$, and θ is an adjustment parameter to adjust the influence of the shortest distance between nodes. In an unweighted network, the shortest distance between two nodes is defined as the number of edges on the shortest path connecting the two nodes, while in a weighted network, the shortest distance between two nodes is the sum of the weights of the edges on the shortest path.

Based on this, the influence $W\text{\_}\mathrm{LGI}\left(v_{i}\right)$ of the node $v_{i}$ in the network is the product of the node’s local influence $W\text{\_}\mathrm{LI}\left(v_{i}\right)$ and global influence $W\text{\_}\mathrm{GI}\left(v_{i}\right)$, as shown in Equation (9). Calculate the influence of each node in the network, and sort according to the value of the calculated influence from high to low, the order of the importance of the nodes in the network can be obtained, so the identification of influential nodes in the network can be realized.

(9) $$W\text{\_}\mathrm{LGI}\left(v_{i}\right)=W\text{\_}\mathrm{LI}\left(v_{i}\right)\times \;W\text{\_}\mathrm{GI}\left(v_{i}\right)=\frac{d_{w}\left(v_{i}\right)}{n}\times \underset{i\neq j}{\sum }\frac{\sqrt{d_{w}\left(v_{j}\right)}}{d_{\mathrm{ij}}+\theta }\;\;$$

## 4. Experiment

### 4.1. Experimental Data

This paper selects 48 cities in the three major urban agglomerations of Beijing-Tianjin-Hebei, Pearl River Delta and Yangtze River Delta as the research areas, and selects the air quality index (AQI) as the measurement indicator. The data is obtained from the China Air Quality Online Monitoring and Analysis Platform, and we select the hourly data of each monitoring station in each city from 1 November 2020 to 31 October 2021 during a postponed year. As a result of processing the data, the daily average AQI value of each city is obtained. The processed data includes 17,520 city data and 4380 monitoring stations data for Beijing, making a total of 21,900 pieces of data, of which 18 pieces of missing data need to be filled. Due to the continuity of air quality, the missing data can be replaced by averaging the previous day’s value and the next day’s value [50]. At the same time, the amount of missing data is so small that its impact on the spatial correlation of air pollution between regions can be ignored [46]. In addition, the latitude and longitude of each city in this study comes from the search engine.

### 4.2. Analysis of Network Topology Characterization of the Air Pollution Spatial Correlation Weighted Networks in Three Major Urban Agglomerations

**Figure 2:**
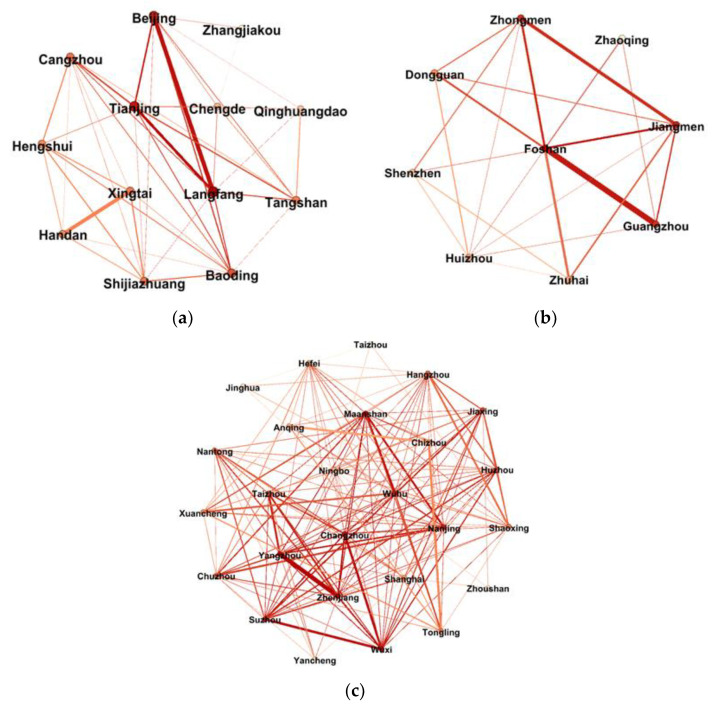
The air pollution spatial correlation weighted networks of the three urban agglomerations: (**a**) Beijing-Tianjin-Hebei; (**b**) Pearl River Delta; and (**c**) Yangtze River Delta.

According to the construction method of the air pollution spatial correlation weighted network proposed in Section 3.1, the air pollution spatial correlation weighted networks of the three major urban agglomerations of Beijing-Tianjin-Hebei, Pearl River Delta, and Yangtze River Delta are constructed, respectively. Taking the Beijing-Tianjin-Hebei urban agglomeration as an example, the 13 cities in the Beijing-Tianjin-Hebei region are taken as nodes. By calculating the Pearson correlation coefficient of daily average AQI values among the 13 cities and averaging them, the mean value of the correlation coefficient of this network is 0.7538, so 0.7538 is used as the threshold to construct edges between the node cities. That is, if the correlation coefficient between the two cities is greater than or equal to 0.7538, there is an edge. Otherwise no edge is established. Then, we calculated the distance between two cities according to the longitude and latitude of the cities, get the distance coefficient between two cities after normalization and divided the correlation coefficient by the distance coefficient to obtain the weights of the edges. So, an undirected weighted network with 13 nodes and 43 edges is constructed. Using the Gephi visualization tool, the air pollution spatial correlation weighted networks of the three major urban agglomerations of Beijing-Tianjin-Hebei, Pearl River Delta, and Yangtze River Delta are drawn, as shown in Figure 2.

It can be seen from Figure 2 that there are no isolated city nodes in the networks, which means that in the face of the spatial correlation network of air pollution, no city can be alone and all will be affected by other cities. According to the calculation method of the overall network structural characteristics proposed in Section 3.2, Table 1 reports the calculation results of the structural characteristics indicators of the three major urban agglomeration networks. The air pollution spatial correlation networks of three major urban agglomerations of Beijing-Tianjin-Hebei, Pearl River Delta, and Yangtze River Delta are all relatively close, and the network density of the networks of the three urban agglomerations exceeds 0.5, indicating that the spatial correlation of air pollution among cities is relatively high, among which the network density of the Pearl River Delta is the highest, reaching 0.639 and the Beijing-Tianjin-Hebei and Yangtze River Deltas are slightly lower than the Pearl River Delta. The three major urban agglomerations all show a multi-city, multi-threaded, and cross-regional network distribution. The network efficiency of the networks of the three major urban agglomerations is all around 0.5, and there are many spillover channels of air pollution between cities, indicating that the spatial correlation structure of air pollution in the three major urban agglomerations is relatively stable, but there is still a lot of room to strengthen the connection between cities. The network rank degree of the three major urban agglomerations is relatively high, all of them exceeding 0.9, indicating that the three major urban agglomerations have a relatively high air pollution rank attribute and have significant asymmetric spatial spillover effects. The cities in the networks belong to the core and edge positions. Therefore, it is necessary to further mine and identify influential nodes in the networks.

### 4.3. Influential Nodes Identification of Air Pollution Spatial Correlation Weighted Networks in Three Urban Agglomerations

According to the influential nodes identification method proposed in Section 3.3 of this paper, the attribute values of each node in the three major urban agglomeration networks of Beijing-Tianjin-Hebei, Pearl River Delta, and Yangtze River Delta can be obtained, as shown in Table 2.

**Figure 3:**
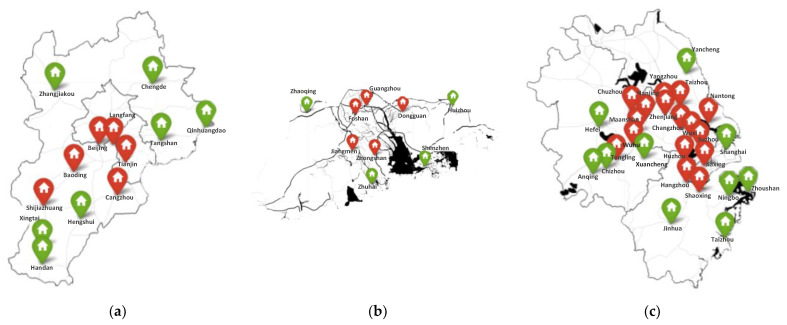
The location of each node in the networks of the three major urban agglomerations: (**a**) Beijing-Tianjin-Hebei; (**b**) Pearl River Delta; and (**c**) Yangtze River Delta.

Based on the above calculations, the influence rankings of the nodes of the air pollution spatial correlation networks in the three major urban agglomerations of Beijing-Tianjin-Hebei, Pearl River Delta, and Yangtze River Delta are obtained. The influence ranking of nodes in the Beijing-Tianjin-Hebei urban agglomeration is: Langfang, Tianjin, Beijing, Baoding, Shijiazhuang, Cangzhou, Tangshan, Handan, Xingtai, Hengshui, Chengde, Qinhuangdao, Zhangjiakou; the influence ranking of nodes in the Pearl River Delta urban agglomeration is: Foshan, Jiangmen, Zhongshan, Guangzhou, Dongguan, Huizhou, Zhuhai, Shenzhen, Zhaoqing; the influence ranking of nodes in the Yangtze River Delta urban agglomeration is: Zhenjiang, Nanjing, Wuxi, Yangzhou, Maanshan, Changzhou, Suzhou, Wuhu, Jiaxing, Huzhou, Taizhou, Hangzhou, Chuzhou, Shaoxing, Nantong, Tongling, Chizhou, Shanghai, Xuancheng, Hefei, Ningbo, Anqing, Yancheng, Zhoushan, Jinhua, Taizhou. In the Beijing-Tianjin-Hebei urban agglomeration, the W_LGI values of the top six cities are all over 2, and they are relatively central in the network; while the W_LGI values of the last seven cities are all below 2, they are relatively edge in the network. In the Pearl River Delta urban agglomeration, the W_LGI values of the top 5 cities are all over 1.3, and they are relatively central in the network; while the W_LGI values of the last 4 cities are all below 1.3, they are relatively edge in the network. In the Yangtze River Delta urban agglomeration, the W_LGI values of the top 16 cities are all over 3, and they are relatively central in the network; the W_LGI values of the bottom 10 cities are all lower than 3, and they are relatively edge in the network. Figure 3 shows the relative positions of the cities in the three major urban agglomerations with the more influential node cities marked in red and the less influential node cities marked in green. It can be seen that the cities that are relatively central in the networks are also relatively centered in terms of geographical location, and have a greater impact on other nodes cities in the region. Cities in relatively edge positions in the network are also in “marginal positions” geographically, and their impact on other cities in the region in terms of air pollution is relatively low, which verifies the hypothesis proposed in this paper.

### 4.4. Analysis of Air Pollution Spatial Correlation Weighted Network within Influential City

After identifying the influential cities that are at the core of the spatial correlation networks based on the urban agglomerations, in order to provide assistance to environmental managers to obtain targeted measures it is necessary to further refine the study area of the spatial correlation of air pollution. Further analysis can be carried out within influential cities. According to the construction method of the air pollution spatial correlation weighted network proposed in Section 3.1, the air pollution spatial correlation weighted network of a certain influential city is constructed with the air quality monitoring stations of the city as nodes. Then, according to the influential nodes identification method proposed in Section 3.3 mining and identifying the monitoring stations at the core of the network in the influential city can help to quickly locate small areas with high pollution levels and high transmission capacity, thus improving the accuracy and efficiency of environmental management efforts.

**Figure 4:**
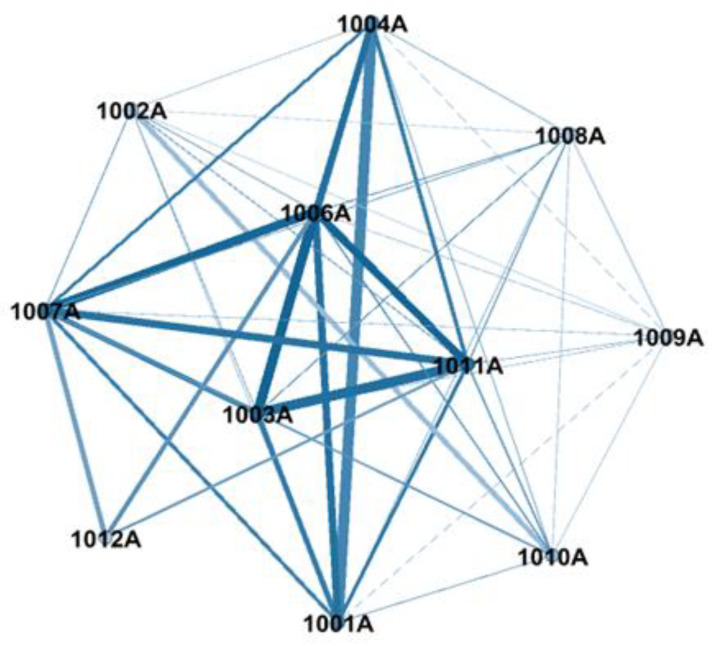
The air pollution spatial correlation weighted network of Beijing.

Taking Beijing, the influential city with the higher rank of influential nodes in the air pollution spatial correlation weighted network of the Beijing-Tianjin-Hebei urban agglomeration, as an example, there are a total of 12 monitoring stations, as shown in Table 3. During the network construction process, the monitoring station “1005A” is not connected to any other node, so this monitoring station is excluded. From this, an air pollution spatial correlation weighted network with 11 nodes and 48 edges is constructed, as shown in Figure 4. After calculation, the network density of this network is 0.727, indicating that there is a very close spatial correlation between the areas where the monitoring stations are located within the city; the network efficiency is 0.156, indicating that the dynamic correlation between air pollution has strong network stability; the network rank degree is 0.981, indicating that the air quality monitoring stations in the city belong to the core and edge positions.

**Figure 5:**
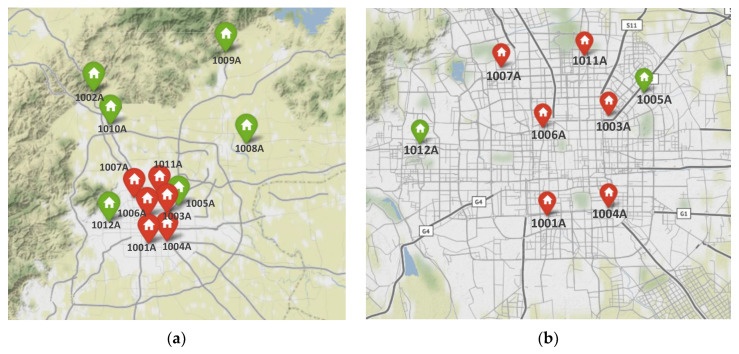
Maps of the locations of the 12 air quality monitoring stations in Beijing: (**a**) map of all areas of Beijing; (**b**) map of urban areas of Beijing.

According to the influential nodes identification method proposed in Section 3.3, the influential nodes are identified for the air pollution spatial correlation weighted network in the influential city of Beijing, and the results are shown in Table 4. According to the results, the top six monitoring stations—“Guanyuan”, “Dongsi”, “Aotizhongxin”, “Tiantan”, “Haidianquwanliu”, and “Wanshouxigong” are all of high influence in the network, with $W\text{\_}\mathrm{LGI}\left(v_{i}\right)$ of 4.5 or more, while the bottom six monitoring stations —“Changpingzhen”, “Dingling”, “Shunyixincheng”, “Huairouzhen”, “Gucheng” and “Nongzhanguan” are less important in the network, with $W\text{\_}\mathrm{LGI}\left(v_{i}\right)$ below 3, which are the less important positions in the network. Figure 5 shows the location of the 12 monitoring stations on the map of Beijing with the top six monitoring stations in terms of influence are marked in red and the last six monitoring stations are marked in green. It can be seen that the top six monitoring stations are all located in the main urban areas of Beijing. These areas have high pedestrian flow and frequent traffic jams, resulting in serious pollutant emissions and a high degree of air quality correlation, and are among the areas in the air pollution network in Beijing that need to be focused on strengthening prevention and control. While among the bottom six monitoring stations, all monitoring stations except “Nongzhanguan” and “Gucheng” are located in the suburbs of Beijing, which are affected by many factors such as distance, pollutant emissions and terrain, and cause relatively low air pollution to other areas in the region. In general, the air pollution in Beijing has a complex spatial inter-pollution phenomenon.

## 5. Conclusions and Suggestions

### 5.1. Conclusions

Based on the complex network theory, this paper studies and analyzes the air pollution spatial correlation weighted networks in the three major urban agglomerations of Beijing-Tianjin-Hebei, Pearl River Delta, and Yangtze River Delta, and draws the following conclusions:

1. By constructing three spatial correlation weighted networks of air pollution in the three major urban agglomerations with cities as nodes and a spatial correlation weighted network of air pollution within a city with air quality monitoring stations as nodes, the network density of each network exceeds 0.5. It can be found that there is a general correlation of air pollution within and between cities and this correlation has transcended the limitation of geographical distance and is intertwined, showing a multi-threaded complex network distribution situation with strong links;
2. Based on the air pollution spatial correlation weighted networks in the three urban agglomerations of Beijing-Tianjin-Hebei, Pearl River Delta, and Yangtze River Delta, the influence of each node city in each network is obtained by using the influential nodes identification method. In the Beijing-Tianjin-Hebei urban agglomeration, cities such as Langfang, Tianjin, Beijing and Baoding occupy the important positions in the network; in the Pearl River Delta urban agglomeration, cities such as Foshan, Jiangmen and Zhongshan are relatively central in the network; in the Yangtze River Delta urban agglomeration, cities such as Zhenjiang, Nanjing, Wuxi, Yangzhou, and Maanshan occupy the important positions in the network. These cities have a strong ability to control the spread of air pollution in other cities and are most closely related to air pollution in other cities, so they need to be the focus of attention in the management of air pollution;
3. Using the influence rankings of cities based on influential nodes in the air pollution spatial correlation weighted networks of the urban agglomerations, the influential cities in the networks are further studied and analyzed. This paper takes Beijing, an influential city of the Beijing-Tianjin-Hebei urban agglomeration, as an example. We evaluate the influence of 12 air quality monitoring stations in the network based on the spatial correlation weighted network of air pollution in Beijing, and conclude that monitoring stations such as “Guangyuan”, “Dongsi”, “Aotizhongxin”, and “Haidianquwanliu” are relatively central in the network. These monitoring stations are also located in the main urban areas of Beijing in terms of spatial distribution, and have the relatively strong ability to spread air pollution to the surrounding areas.

### 5.2. Policy Suggestions

Based on complex network theory, this paper studies and analyzes the network structure characteristics of air pollution in China’s three major urban agglomerations—Beijing-Tianjin-Hebei, Pearl River Delta, and Yangtze River Delta. The spatial correlation network structure of air pollution provides new ideas for the construction of a collaborative air pollution governance system, and therefore the following suggestions are proposed.

1. Set up regional joint supervision departments, make overall planning according to regional characteristics, and formulate joint prevention and control mechanisms. In the face of the correlation network and network structure of air pollution in urban agglomerations, no city within an urban agglomeration can be left alone in terms of air quality. Even if a city makes efforts to combat air pollution, which may result in a slight improvement in local air quality in the short term, the spatial correlation network of air pollution will quickly offset its efforts. Therefore, taking the lead in the joint prevention and control of air pollution within the urban agglomeration, and then constructing a cross-regional joint prevention and control system is an inevitable choice to solve the problem of air pollution as a whole;
2. For the influential cities in the urban agglomeration networks and the influential monitoring stations in the city network, these nodes are relatively central in the network and have a strong spatial spillover effect in terms of air pollution. Therefore, it is necessary to intensify its monitoring efforts, establish key monitoring mechanisms, and formulate measures (such as formulating more stringent energy-saving and emission reduction policies, etc.) to cut off or weaken the transmission channels of air pollution between these areas and other areas, so as to reduce the connectivity efficiency of the entire air pollution spatial correlation network, thereby achieving the effect of reducing air pollution in the whole region;
3. Further improve policies and regulations on air pollution prevention and control, and introduce more scientific collaborative governance plans. In order to effectively respond to the spatial correlation of air pollution, attempts can be made to break through the constraints of the traditional administrative region system and achieve cross-regional and cross-departmental collaboration, with greater synergy between regions in controlling population size, urban investment intensity and industrial emission reduction. In the end, while maximizing the effect of collaborative pollution control, it will achieve a comprehensive range of regional synergistic development in a wider spatial context, innovate green production methods and promote the realization of the “carbon peak and carbon neutrality” goals of each city and region;
4. Formulate and implement specific measures for the collaborative governance of air pollution. First of all, according to the main origin of air pollution in different regions, special attention should be paid to optimize the spatial layout of industrial enterprises in the region as a whole. By strictly controlling industries with high pollution and high energy consumption, the transmission and transfer of air pollution in the region should be strictly prevented. Strengthen the unified control of motor vehicle pollution emissions by means of improving the regional transportation system and raising the emission level. Secondly, by flexibly using taxation, subsidies, credit and other economic means, as well as regulatory and punitive measures for enterprises, activate the enthusiasm of enterprises in various regions to participate in air pollution control and explore more reasonable behavioral strategies for emission reduction, so as to provide an important path to achieve the “carbon peak and carbon neutrality” goals. In addition, advanced technologies such as big data are used to integrate various city systems and establish a comprehensive, unified and efficient big data information platform in the region.

In conclusion, the prevention and control of air pollution in various urban agglomerations and regions in China is a complex systematic project. This paper provides new ideas for the air pollution prevention and control from the perspective of the spatial correlation of air pollution. It is important to re-examine and build cross-regional collaborative governance strategies from a global perspective, pay attention to the spatial linkage effect within the urban agglomerations, and play the role of central nodes. In this way, we can better improve the efficiency and effectiveness of air pollution governance, jointly achieve green development and help achieve the “carbon peak and carbon neutrality” goals.

## Figures and Tables

**Table 1:** Structural characteristics of air pollution spatial correlation weighted networks of three urban agglomerations of Beijing-Tianjin-Hebei, Pearl River Delta, and Yangtze River Delta.

	Number of Nodes (n)	Number of Edges (m)	Network Density (G_d_)	Network Efficiency (G_e_)	Network Rank Degree (G_r_)
Beijing-Tianjin-Hebei	13	43	0.551	0.531	0.911
Pearl River Delta	9	23	0.639	0.476	0.992
Yangtze River Delta	26	165	0.508	0.536	0.934

**Table 2:** The attribute values of each node in the three major urban agglomeration networks: (**a**) Beijing-Tianjin-Hebei; (**b**) Pearl River Delta; and (**c**) Yangtze River Delta.

	${d(v}_{i})$	$d_{w}{(v}_{i})$	${W\text{\_}\mathrm{LI}(v}_{i})$	${W\text{\_}\mathrm{GI}(v}_{i})$	${W\text{\_}\mathrm{LGI}(v}_{i})$	Rank
(**a**)						
Beijing	8	2.601	0.200	14.170	2.834	3
Tianjin	9	2.720	0.209	14.392	3.008	2
Shijiazhuang	8	1.778	0.137	15.065	2.064	5
Baoding	9	2.133	0.164	14.488	2.376	4
Tangshan	7	1.720	0.132	14.530	1.918	7
Langfang	8	2.933	0.226	14.171	3.203	1
Qinhuangdao	5	0.891	0.068	14.941	1.016	12
Zhangjiakou	2	0.299	0.023	14.043	0.323	13
Handan	5	1.579	0.121	14.322	1.733	8
Hengshui	6	1.597	0.123	13.886	1.708	10
Cangzhou	8	1.819	0.140	14.728	2.062	6
Chengde	6	1.079	0.083	14.542	1.207	11
Xingtai	5	1.786	0.137	13.781	1.888	9
(**b**)						
Guangzhou	5	2.036	0.226	8.743	1.978	4
Shenzhen	4	0.899	0.100	9.006	0.899	8
Zhuhai	4	1.172	0.131	9.207	1.199	7
Dongguan	5	1.402	0.156	8.646	1.347	5
Foshan	6	2.384	0.265	8.774	2.324	1
Zhongshan	6	2.026	0.225	8.825	1.986	3
Huizhou	7	1.125	0.125	10.104	1.263	6
Jiangmen	7	2.132	0.237	9.038	2.141	2
Zhaoqing	2	0.488	0.054	8.395	0.456	9
(**c**)						
Shanghai	13	1.767	0.068	38.689	2.629	18
Nanjing	20	3.465	0.133	38.834	5.176	2
Wuxi	17	3.487	0.134	37.861	5.078	3
Changzhou	17	3.297	0.127	37.952	4.813	6
Suzhou	16	3.206	0.123	37.799	4.661	7
Nantong	13	2.134	0.082	37.856	3.108	15
Yancheng	6	0.749	0.029	37.706	1.086	23
Yangzhou	15	3.469	0.133	37.517	5.006	4
Zhenjiang	17	3.771	0.145	38.164	5.535	1
Taizhou	13	2.669	0.103	36.753	3.773	11
Hangzhou	17	2.409	0.093	38.963	3.610	12
Ningbo	12	1.628	0.063	38.115	2.386	21
Jiaxing	16	2.723	0.105	38.117	3.993	9
Huzhou	15	2.634	0.101	37.543	3.808	10
Shaoxing	15	2.092	0.080	39.143	3.150	14
Jinhua	4	0.473	0.018	36.987	0.673	25
Zhoushan	3	0.551	0.021	35.639	0.756	24
Taizhou	3	0.353	0.014	36.554	0.496	26
Hefei	13	1.642	0.063	38.709	2.444	20
Wuhu	17	3.093	0.119	38.365	4.564	8
Maanshan	18	3.361	0.129	38.218	4.941	5
Tongling	10	2.123	0.082	37.646	3.073	16
Anqing	8	1.430	0.055	38.178	2.099	22
Chuzhou	14	2.306	0.089	37.740	3.348	13
Chizhou	8	1.843	0.071	37.474	2.657	17
Xuancheng	10	1.817	0.070	37.169	2.597	19

Note: In the tables,${\;d(v}_{i})$ represents the degree of node $v_{i}$, $d_{w}\left(v_{i}\right)$ represents the weighted degree of node $v_{i}$, $W\text{\_}\mathrm{LI}\left(v_{i}\right)$ represents the local influence of node $v_{i}$, ${W\text{\_}\mathrm{GI}(v}_{i})$ represents the global influence of node $v_{i}$, ${W\text{\_}\mathrm{LGI}(v}_{i})$ represents the comprehensive influence of the node $v_{i}$, that is, the influence score, and Rank is the influence ranking of the node.

**Table 3:** Information on air quality monitoring stations in Beijing.

Station Number	Station Name	Latitude (°N)	Longitude (°E)	Station Type
1001A	Wanshouxigong	39.867	116.366	Urban
1002A	Dingling	40.286	116.170	Suburban
1003A	Dongsi	39.952	116.434	Urban
1004A	Tiantan	39.874	116.434	Urban
1005A	Nongzhanguan	39.972	116.473	Urban
1006A	Guanyuan	39.942	116.361	Urban
1007A	Haidianquwanliu	39.993	116.315	Urban
1008A	Shunyixincheng	40.144	116.720	Suburban
1009A	Huairouzhen	40.394	116.644	Suburban
1010A	Changpingzhen	40.195	116.230	Suburban
1011A	Aotizhongxin	40.003	116.407	Urban
1012A	Gucheng	39.928	116.22	Urban

**Table 4:** Ranking of influential nodes in the air pollution spatial correlation weighted network of Beijing.

Station Number	Rank	$W\text{\_}\mathrm{LGI}\left(v_{i}\right)$	Station Type
1001A	6	4.764	Urban
1002A	8	2.298	Suburban
1003A	2	5.582	Urban
1004A	5	4.795	Urban
1005A	12	0	Urban
1006A	1	6.435	Urban
1007A	4	5.314	Urban
1008A	9	2.202	Suburban
1009A	10	1.667	Suburban
1010A	7	2.658	Suburban
1011A	3	5.580	Urban
1012A	11	1.415	Urban

## Data Availability

The data used to support the findings of this study are available from the corresponding author upon request.
